# Regional Differences of Undiagnosed Type 2 Diabetes and Prediabetes Prevalence Are Not Explained by Known Risk Factors

**DOI:** 10.1371/journal.pone.0113154

**Published:** 2014-11-17

**Authors:** Teresa Tamayo, Sabine Schipf, Christine Meisinger, Michaela Schunk, Werner Maier, Christian Herder, Michael Roden, Matthias Nauck, Annette Peters, Henry Völzke, Wolfgang Rathmann

**Affiliations:** 1 Institute of Biometrics and Epidemiology, German Diabetes Center, Leibniz Center for Diabetes Research at Heinrich-Heine-University, Düsseldorf, Germany; 2 Institute for Community Medicine, University Medicine Greifswald, Greifswald, Germany; 3 Helmholtz Zentrum München, German Research Center for Environmental Health, Institute of Epidemiology II, Neuherberg, Germany; 4 Helmholtz Zentrum München, German Research Center for Environmental Health, Institute of Health Economics and Health Care Management, Neuherberg, Germany; 5 Institute for Clinical Diabetology, German Diabetes Center, Leibniz Center for Diabetes Research at Heinrich-Heine-University, Düsseldorf, Germany; 6 Department of Endocrinology and Diabetology, University Hospital Düsseldorf, Düsseldorf, Germany; 7 Institute of Clinical Chemistry and Laboratory Medicine, University Medicine Greifswald, Greifswald, Germany; 8 German Center for Diabetes Research (DZD e.V.), Sites Düsseldorf and Munich, Germany; 9 German Center of Cardiovascular Research (DZHK e.V.), Sites Munich and Greifswald, Germany; 10 Competence Network Diabetes mellitus (Federal Ministry of Education and Research, Germany), Sites Düsseldorf, Munich and Greifswald, Germany; University of Michigan Medical School, United States of America

## Abstract

**Background:**

We have previously found regional differences in the prevalence of known type 2 diabetes between northeastern and southern Germany. We aim to also provide prevalence estimates for prediabetes (isolated impaired fasting glucose (i-IFG), isolated glucose intolerance (i-IGT), combined IFG and IGT) and unknown type 2 diabetes for both regions.

**Methods:**

Prevalence (95%CI) of prediabetes (i-IFG: fasting glucose 5.6–6.9 mmol/l; i-IGT: 2 h postchallenge gluose 7.8–11.0 mmol/l, oral glucose tolerance test (OGTT), ≥8 h overnight fasting) and unknown diabetes were analyzed in two regional population-based surveys (age group 35–79 years): SHIP-TREND (Study of Health in Pomerania (northeast), 2008–2012) and KORA F4 (Cooperative Health Research in the region of Augsburg (south), 2006–2008). Both studies used similar methods, questionnaires, and identical protocols for OGTT. Overall, 1,980 participants from SHIP-TREND and 2,617 participants from KORA F4 were included.

**Results:**

Age-sex-standardized prevalence estimates (95%CI) of prediabetes and unknown diabetes were considerably higher in the northeast (SHIP-TREND: 43.1%; 40.9–45.3% and 7.1%; 5.9–8.2%) than in the south of Germany (KORA F4: 30.1%; 28.4–31.7% and 3.9%; 3.2–4.6%), respectively. In particular, i-IFG (26.4%; 24.5–28.3% vs. 17.2%; 15.7–18.6%) and IFG+IGT (11.2%; 9.8–12.6% vs. 6.6%; 5.7–7.5%) were more frequent in SHIP-TREND than in KORA. In comparison to normal glucose tolerance, the odds of having unknown diabetes (OR, 95%CI: 2.59; 1.84–3.65) or prediabetes (1.98; 1.70–2.31) was higher in the northeast than in the south after adjustment for known risk factors (obesity, lifestyle).

**Conclusions:**

The regional differences of prediabetes and unknown diabetes are in line with the geographical pattern of known diabetes in Germany. The higher prevalences in the northeast were not explained by traditional risk factors.

## Introduction

According to the sixth edition of the IDF Diabetes atlas [1], considerable regional differences in the prevalence of type 2 diabetes (age-adjusted) exist between European nations ranging from 2.4% in Moldova to more than 14% in Turkey [2]. While geographic variations in diabetes prevalence between nations are well known, regional differences within countries are rarely analyzed. Especially, information on regional variation of prediabetes and unknown (undiagnosed) diabetes is lacking. In the USA, the Behavioral Risk Factor Surveillance System identified higher prevalence estimates of known (diagnosed) type 2 diabetes of 11.7% in the adult population of the southeastern area (“diabetes belt”) while the comparative estimate was 8.5% for the rest of the country [3]. In a meta-analysis of regional surveys in the Diabetes Collaborative Research of Epidemiologic Studies (DIAB-CORE consortium) we found a high prevalence of known type 2 diabetes in the northeastern regions of Germany with 10.9% in Pomerania (Vorpommern) and a comparatively low prevalence of 5.8% in the south [4]. However, it remained unclear whether a higher awareness and screening for the disease among general practitioners and in the population led to a lower frequency of undiagnosed diabetes, or whether a higher morbidity itself may have contributed to higher prevalence estimates in the northeast. Therefore, we compared age- and sex-specific estimates for prediabetes and unknown diabetes in two German regions – one in the northeast (Pomerania, SHIP-TREND) and one in the south (Augsburg region, KORA F4) using oral glucose tolerance tests (OGTT, ADA criteria) [5]. The study sampling and standard operating procedures of SHIP-TREND were largely similar to KORA F4 (including OGTT) in order to provide a high comparability between the two studies.

## Methods

### Study population SHIP-TREND (northeastern Germany)

A sample of 8,826 adults aged 20–79 years with German nationality was drawn from the central population registry of Western Pomerania (212,157 inhabitants living in Greifswald, Stralsund, Anklam and surrounding counties). A two-stage cluster sampling method was adopted from the WHO Multinational Monitoring of Trends and Determinants in Cardiovascular Disease (MONICA) Project in Augsburg, Germany. Stratification variables were age, sex, and city/county of residence [6]. Of all persons invited, 4,420 (50.1%) individuals participated in the examinations (2008–2012). All participants provided written informed consent and the medical ethics committee of the University of Greifswald approved the study protocol. Further information on the study design of the SHIP-TREND survey has been published elsewhere [6], [7].

### Study population KORA F4 (southern Germany)

The KORA F4 study is the follow-up of the KORA S4 survey which was carried out in 1999–2001 in the city of Augsburg and 16 municipalities from the surrounding counties (about 600,000 inhabitants). The re-investigation took place in 2006–2008 (KORA F4). A two-stage cluster sampling method was used which followed the WHO MONICA project method in the Augsburg region. For Augsburg city, a simple random sampling was performed. In the surrounding rural districts, 16 counties were selected with probabilities proportional to their size [8]. Of 6,640 participants with German nationality aged 25–74 years, 4,261 participated at the baseline examinations S4. In KORA F4, 3,080 (72%) S4 participants were re-investigated. At baseline, an oral glucose tolerance test (OGTT) was administered to all 1,353 non-diabetic subjects in the age group 55–74 years [9]. Younger participants were not screened for undiagnosed diabetes. At follow-up, OGTT measurements were carried out without age restriction after exclusion of individuals with known diabetes [9], [10]. For the current study, the cross-sectional data of KORA F4 was analyzed only. The OGTT-based screening for unknown diabetes in KORA S4 in age group 55–74 years at baseline, (62–82 years at follow-up) might have resulted in an early diagnosis of diabetes and therefore in a lower number of unknown diabetes at follow-up. Because of this methodological difference to SHIP-TREND, we report results for younger (<60 years) and older participants separately. All study participants gave written informed consent to the study, which was approved by the Ethics Committee of the Bavarian Medical Association.

### Laboratory analyses including glucose measurements

In both studies, blood was collected without stasis after an overnight fast of ≥8 hours. After blood withdrawal the blood samples were immediately centrifuged and kept cool (4°C) until analysis of blood glucose in the central laboratory. Measurements of fasting glucose and 2 h glucose were based on plasma in SHIP-TREND and on serum in KORA F4. In order to determine whether both samples were comparable, duplicate measurements were carried out using serum samples of all SHIP-TREND participants. Both measurements (plasma and serum glucose) were highly correlated (r = 0.99; p<0.0001). In Passing Bablok regression analysis of serum versus plasma glucose (mmol/l) an intercept of −0.10 (95%CI −0.10; −0.10) and a slope of 1 were observed concluding that plasma values in SHIP-TREND might be only slightly lower than serum values. Furthermore, 30 serum blood glucose samples were drawn at random from KORA F4 and re-assessed in the SHIP-TREND laboratory, yielding a correlation coefficient of r = 0.94 (p<0.0001). On average, these original 30 KORA F4 measurements were only slightly lower (mean −0.06 mmol/l; SD 0.17) when they were re-analyzed in the SHIP-TREND laboratory. Thus, serum glucose from KORA F4 and plasma glucose from SHIP-TREND were considered as comparable for the current analysis.

HDL-, LDL- and total cholesterol, and triglycerides were measured as described elsewhere [6], [11].

### Ascertainment of diabetes and prediabetes in SHIP-TREND and KORA F4

Oral glucose tolerance tests followed concordant standardized operating procedures (SOP) in both studies [9]. Participants were asked to fast for ≥8 hours except for water (eg. from 22∶00 p.m.) and should not smoke or consume caffeine containing drinks, such as coffee and avoid sports before the examination. In SHIP-TREND and KORA F4, OGTTs were completed during morning hours. Fasting glucose was sampled, and 75 grams of anhydrous glucose (Dextro OGT; Boehringer Mannheim, Mannheim Germany) was given to participants without known diabetes (SHIP-TREND: known diabetes or current use of glucose-lowering agents; KORA F4: current use of glucose-lowering agents or unknown (newly study-diagnosed) diabetes at baseline (S4), validated by a physician). Fasting glucose and 2 h postload glucose were measured using a hexokinase method. In SHIP-TREND, Dimension Vista by Siemens Healthcare Diagnostics (Eschborn, Germany) and in KORA F4, GLU flex by Dade Behring (Marburg, Germany) were used.

Participants with fasting glucose values <5.6 mmol/l (<100 mg/dl) and 2 h glucose <7.8 mmol/l (<140 mg/dl) were defined as having normal glucose tolerance (NGT) (5). Fasting glucose values ≥7.0 mmol/l (≥126 mg/dl) or 2 h glucose ≥11.1 mmol/l (≥200 mg/dl) were classified as unknown diabetes. Prediabetes was diagnosed if fasting glucose values ranged between 5.6 and 6.9 mmol/l (100–125 mg/dl, i-IFG) and/or 2 h postload glucose values between 7.8 and 11.0 mmol/l (140–199 mg/dl, i-IGT). Participants were categorized to three groups of prediabetes (glucose disturbances): isolated impaired fasting glucose (i-IFG), isolated impaired glucose tolerance (i-IGT), and combined IFG and IGT (IFG+IGT).

### Anthropometry, interviews and medical information in SHIP-TREND and KORA F4

In both studies, trained and certified personnel collected information on sociodemographic variables, lifestyle habits and medical history by the following common standardized personal interviews. Participants were asked to bring original packaging of their medications that were taken during the last 7 days before the examination date. Unique identifiers and drug names were recorded according to the Anatomical Therapeutical Chemical (ATC) classification system.

Trained staff measured height and weight of participants wearing lightweight clothing without shoes. Blood pressure was measured in sitting position using a validated automatic device (SHIP-TREND and KORA F4: OMRON HEM 705-CP). Three independent measurements were taken in three-minute-intervals after an initial rest of at least 5 minutes. The mean of the second and third blood pressure measurement was calculated in both studies and used for the analysis.

### Lifestyle and socioeconomic indicators

Physical activity level was based on self-reported time per week spent on sports during leisure time in summer and winter. Low exercise level activities such as stepping stairs or walking were not assessed. Participants were considered as inactive if their leisure time exercise comprised <1 h per week in summer or winter.

Participants were classified as smokers if they smoked at least one cigarette per day and as non-smokers if they were either never smoking or stopped smoking at least 12 month ago. Alcohol consumption (in g/day) was calculated from self-reported consumption of beer, wine, and liquors per week.

Educational level was assessed by highest self-reported schooling degree achieved. Low education was defined as ≤9 years of schooling either with school leaving certificate (completed lower secondary education level) or without.

### Study population

The study is based on participants aged 35 to 79 years (SHIP-TREND: n = 3,624; KORA F4: n = 2,929). Participants were excluded from the analyses of pre-diabetes states if one of the following criteria was met: self-reported, known diabetes, or diabetes treatment (SHIP-TREND: n = 430; KORA F4: n = 225), missing information on known diabetes (SHIP-TREND: n = 12; KORA F4: n = 86), or missing or implausible values in glucose measurements (SHIP-TREND: n = 536; KORA = 1).

All participants who underwent glucose challenge testing were required to be in the fasting state (≥8 hours overnight fasting). In SHIP-TREND, however, 666 participants without known diabetes were not fasting for 8 hours. Therefore, estimates for known diabetes are reported for the complete sample including non-fasting participants (SHIP-TREND: n = 3,624; KORA F4: n = 2,928). For the estimation of unknown diabetes or prediabetes, participants who did not meet fasting criteria were excluded from analysis. These “non-fasting” 666 participants from SHIP-TREND had a lower proportion of women (43.5% vs. 54.6%; p<0.0001), lower HDL-cholesterol (1.4 mmol/l vs. 1.5 mmol/l; p = 0.001) and higher triglycerides (median 1.6 vs. 1.3 mmol/l; p<0.0001) than the 1,980 participants with 8 h overnight fasting. However, both groups from SHIP-TREND – fasting versus non-fasting participants – did not differ in age, body mass index, total cholesterol, LDL-cholesterol, blood pressure, fasting and 2 h glucose. Therefore, the additional analyses on regional differences in known diabetes include all 3,491 participants from SHIP-TREND and 2,928 participants from KORA F4, whereas the main analyses on prediabetes and unknown diabetes are based on 1,980 participants from SHIP-TREND and 2,617 participants from KORA F4.

### Statistical analysis

For descriptive analyses, mean (SD) were calculated for continuous variables and proportions for categorical variables. Differences between study regions were calculated using Wilcoxon-Test for metric and Fisher’s exact test for dichotomous variables. Statistical significance was set at p<0.05. Prevalence estimates for prediabetes and unknown diabetes were directly standardized to the German adult population (31^st^ December 2007). Logistic regression models were carried out to determine factors associated with either unknown diabetes or prediabetes. Models were fitted adjusting for age, sex, BMI, physical activity, smoking, alcohol consumption, and formal schooling. Analyses were performed using SAS statistical software version 9.3 (SAS Institute Inc., Cary, NC, USA). Distribution of fasting glucose and 2 h glucose were plotted with RStudio (*RStudio*, Inc., Boston, USA) using the kernel density estimation as a method to estimate the probability density function.

## Results

In the complete sample of both studies including individuals with known diabetes and non-fasting individuals aged 35–79 years, age-sex-standardized prevalence of known diabetes was 11.9% (95%CI: 10.8–13.0%) in the northeast and 6.5% (5.2–7.8%) in the south. Individuals with type 2 diabetes from the northeast were younger (63 vs. 67 years; p<0.0001), had higher fasting glucose levels (8.7 vs. 7.7 mmol/l, p = 0.01), a higher body mass index (32.4 vs. 31.3 kg/m^2^; p = 0.02), higher blood pressure (systolic: 137.8 vs. 132.3 mmHg, p<0.001; diastolic: 78.3 vs. 74.4 mmHg; p<0.001), and higher triglycerides (median: 2.0 vs. 1.6 mmol/l; p = <0.001), but slightly lower cholesterol levels (total: 5.1 vs. 5.3 mmol/l; p = 0.07; HDL-Cholesterol: 1.26 vs. 1.27 mmol/l, p = 0.28; LDL-C: 3.1 vs. 3.3 mmol/l, p = 0.011) (Table S1).

For the analysis of prediabetes and unknown diabetes, participants with known diabetes and with a fasting period of <8 hours were excluded, leaving 1,980 participants from SHIP-TREND (1,080 women; 900 men) and 2,617 participants (1,379 women; 1,239 men) from KORA F4. The characteristics of these participants are shown in Table 1. SHIP-TREND participants were slightly younger, but had a higher body mass index, higher blood pressure values, increased triglycerides and elevated fasting and 2 h glucose. The participants of both regions did not differ in cholesterol levels (HDL-cholesterol, LDL-cholesterol and total cholesterol).

**Table 1:** Characteristics of the two population-based regional surveys: SHIP-TREND (northeast) and KORA F4 (south)*.

	SHIP-TREND	KORA F4	p-value
N	1,980	2,617	
Female sex (%)	54.6	52.7	0.221
Age (years)	54.0 (11.4)	55.5 (12.2)	<0.001
Fasting glucose (mmol/l)	5.6 (0.8)	5.3 (0.6)	<0.001
2 h glucose (mmol/l)	6.7 (2.4)	6.2 (2.1)	<0.001
Body mass index (kg/m^2^)	28.1 (4.8)	27.4 (4.6)	<0.001
Systolic blood pressure (mmHg)	126.6 (17.3)	121.6 (18.3)	<0.001
Diastolic blood pressure (mmHg)	77.6 (9.8)	75.4 (9.8)	<0.001
Total cholesterol (mmol/l)	5.6 (1.1)	5.6 (1.0)	0.901
HDL-cholesterol (mmol/l)	1.5 (0.4)	1.5 (0.4)	0.571
LDL-cholesterol (mmol/l)	3.5 (0.9)	3.5 (0.9)	0.105
Triglycerides (mmol/l), median (IQR)	1.3 (0.9–1.8)	1.3 (0.9–1.8)	<0.001

*Results are means (SD), proportions (%) or median (IQR). P-values are calculated using Wilcoxon-Test for metric and Fisher’s exact test for dichotomous variables.

Abbreviations: SHIP-TREND: Study of Health in Pomerania (2008–2012); KORA F4: Cooperative Health Research in the Region of Augsburg;

The sample was restricted to participants without known diabetes and to participants who were fasting for ≥8 hours.

**Figure 1:**
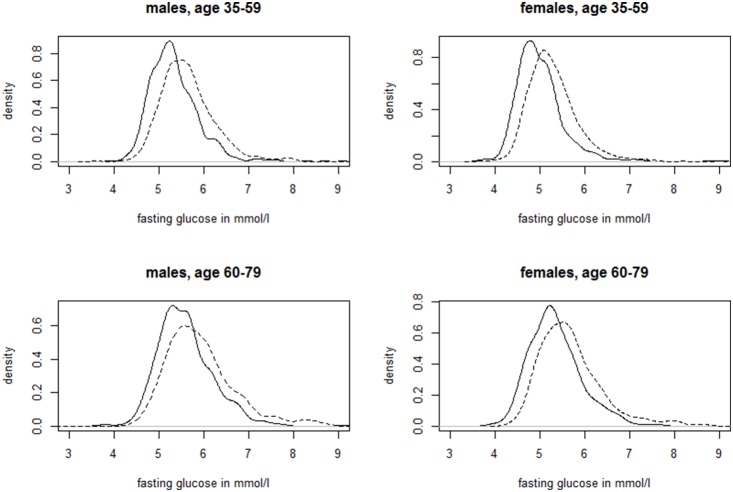
Kernel densities for fasting glucose in mmol/l per study region stratified for age group and sex. Solid line: KORA F4; dashed line: SHIP-TREND.

**Figure 2:**
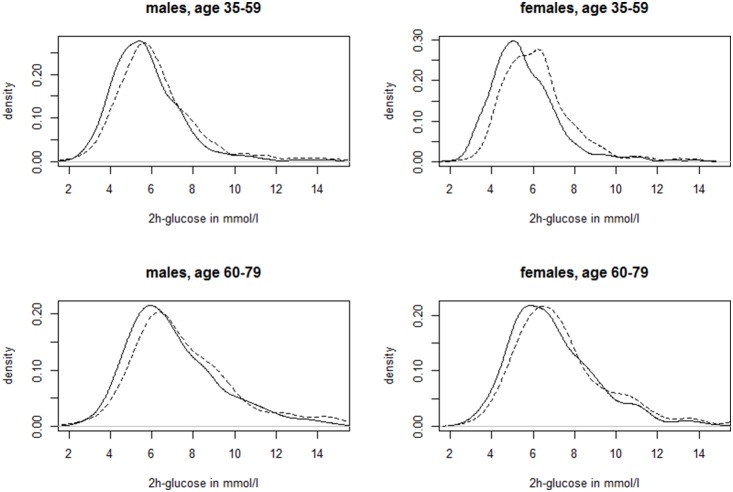
Kernel densities for 2 h glucose in mmol/l per study region stratified for age group and sex. Solid line: KORA F4; dashed line: SHIP-TREND.

The age- and sex-specific distributions of fasting and 2 h glucose values are shown for both study regions in Figures 1 and 2. The distributions were shifted towards generally higher levels both for fasting and 2 h glucose in SHIP-TREND compared to KORA F4. This shift was more pronounced in those with NGT, in particular in the age-group 35–59 years. In this group, the mean difference between the two regional populations for 2 h glucose was 0.3 mmol/l (95%CI: 0.2–0.4 mmol/l). In older individuals with NGT, this mean difference was similar, with slightly wider confidence intervals (0.3; 0.1–0.4 mmol/l). In participants with prediabetes and undiagnosed diabetes this pattern was less clear and differences did not reach statistical significance.

**Figure 3:**
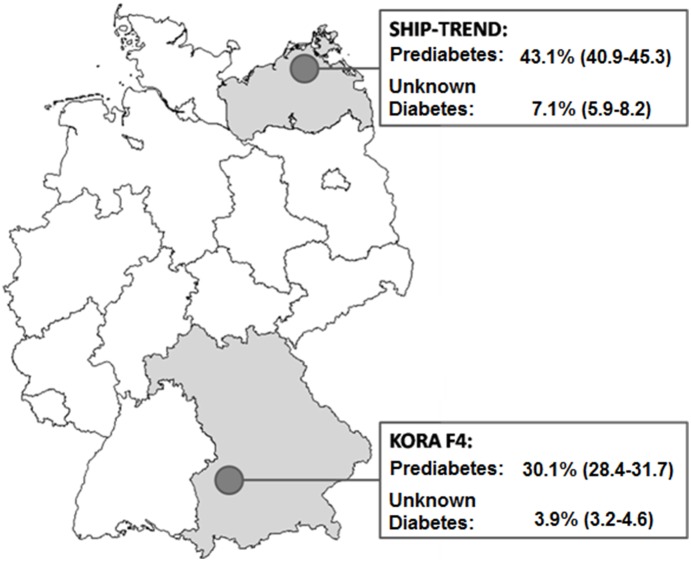
Prevalence of prediabetes (Pre-DM  =  i-IFG, i-IGT or combined IFG+IGT) and unknown diabetes (New-DM) in two German regions, standardized to the German population (31/12/2007).

After age- and sex-standardization to the German population (31.12.2007), the prevalence (95%CI) of unknown diabetes was almost twofold higher in the northeast than in the south (7.1%; 5.9–8.2% vs. 3.9%; 3.2–4.6%). Overall, prediabetes (IFG, IGT or both) was also more prevalent in the northeast (43.0%; 40.8–45.1%) than in the south (30.1%; 28.4–31.7%) (Figure 3).

After stratification for age and sex, the crude estimates of prediabetes were significantly higher in the northeast than in the south in the younger age-groups 35–44 years, 45–54 years, and 55–64 years (Table 2). Prediabetes prevalence was also 2.2-fold higher in males and 2.7-fold higher in women aged 35–44 years. This sex-difference leveled off with increasing age-group and was reversed in men aged 75–79 years, however, not reaching statistical significance.

**Table 2:** Prevalence of prediabetes (i-IFG, i-IGT or combined IFG+IGT) in the northeast (SHIP-TREND) and south (KORA F4) of Germany*.

Age (years)	SHIP-TREND	KORA F4
N	1,980	2,617
**Men**		
35–44	**43.5 (36.8–50.4)**	**20.2 (15.8–25.2)**
45–54	**50.4 (44.0–56.8)**	**35.2 (29.7–41.1)**
55–64	**57.8 (51.2–64.3)**	**44.3 (38.7–50.1)**
65–74	58.2 (50.1–66.0)	52.3 (46.0–58.6)
75–79	43.8 (29.5–58.8)	52.7 (42.1–63.1)
**Women**		
35–44	**19.9 (15.4–25.1)**	**7.3 (4.7–10.6)**
45–54	**30.9 (25.8–36.4)**	**14.7 (11.2–18.9)**
55–64	**45.5 (39.6–51.4)**	**30.7 (25.7–36.1)**
65–74	49.2 (41.7–56.7)	44.1 (38.2–50.0)
75–79	48.5 (30.8–66.5)	41.9 (31.3–52.9)

*The sample was restricted to participants without known diabetes and to participants who were fasting for ≥8 hours. Results are crude prevalence estimates (%) and 95% confidence intervals. Significant regional differences (p<0.05) are highlighted in bold.

Abbreviations: SHIP-TREND: Study of Health in Pomerania (2008–2012); KORA F4: Cooperative Health Research in the Region of Augsburg (2006–2008). I-IFG (isolated impaired fasting glucose: 5.6–6.9 mmol/l (100–125 mg/dl), fasting); i-IGT (impaired glucose tolerance: 7.8–11.0 mmol/l (140–199 mg/dl), 2 hour postload).

With respect to the three subgroups of prediabetes, regional differences in age- and sex-standardized prevalence were significant both for i-IFG and combined IFG+IGT, but did not reach level of statistical significance for i-IGT (SHIP-TREND: i-IFG 26.4% (24.5–28.3%); combined IFG+IGT 11.2% (9.8–12.6%); KORA F4: i-IFG; combined IFG+IGT 17.2% (15.7–18.6%) and 6.6% (5.7–7.5%)).

Table 3 shows age- and sex-specific prevalence estimates for i-IFG, i-IGT, and combined IFG+IGT and unknown diabetes. I-IFG was the most common glucose disturbance in both regions. Prevalence of i-IFG was more than two-fold increased in the northeast in men aged 35–44 years and in women aged 45–54 years compared to the south (Table 3). Among individuals with i-IGT no significant regional differences were found in any age group. In combined IFG and IGT, prevalence was increased in the northeast in comparison to the south in both sexes only in the complete sample aged 35–79 years. Furthermore, prevalence of undiagnosed diabetes was significantly increased in men aged 35–79 years from northeast, while the difference was not significant in women.

**Table 3:** Age- and sex-specific prevalence of glucose disorders in the northeast (SHIP-TREND) and south (KORA F4) of Germany*.

Age (years)	i-IFG		i-IGT		IFG+IGT		Unknown diabetes	
	SHIP-TREND	KORA F4	SHIP-TREND	KORA F4	SHIP-TREND	KORA F4	SHIP-TREND	KORA F4
	n = 526	n = 458	n = 109	n = 173	n = 214	n = 185	n = 140	n = 110
**Men**								
35–44	**34.3 (28.0–41.0)**	**16.5 (12.5–21.2)**	2.8 (1.0–5.9)	1.7 (0.5–3.9)	6.5 (3.6–10.6)	2.0 (0.7–4.3)	2.3 (0.8–5.3)	1.0 (0.2–2.9)
45–54	**38.7 (32.6–45.1)**	**24.6 (19.6–30.0)**	4.0 (2.0–7.3)	5.7 (3.3–9.1)	7.7 (4.7–11.7)	5.0 (2.8–8.2)	5.6 (3.1–9.3)	1.8 (0.6–4.1)
55–64	37.0 (30.7–43.5)	29.1 (24.1–34.5)	3.5 (1.5–6.7)	4.9 (2.7–7.9)	17.4 (12.7–22.9)	10.4 (7.2–14.3)	13.0 (8.9–18.1)	7.4 (4.8–11.0)
65–74	31.0 (23.9–38.8)	28.7 (23.2–34.6)	4.4 (1.8–8.9)	7.0 (4.2–10.8)	22.8 (16.5–30.1)	16.7 (12.3–21.8)	13.9 (8.9–20.3)	7.8 (4.8–11.7)
75–79	18.8 (8.9–32.6)	22.6 (14.6–32.6)	8.3 (2.3–20.0)	16.1 (9.3–25.2)	16.7 (7.5–30.2)	14.0 (7.7–22.7)	16.7 (7.5–30.2)	12.9 (6.8–21.5)
35–79	**34.8 (31.7–38.0)**	**24.5 (22.1–27.0)**	3.9 (2.7–5.4)	5.6 (4.4–7.0)	**13.0 (10.9–15.4)**	**8.7 (7.2–10.4)**	**8.8 (7.0–10.8)**	**5.1 (3.9–6.5)**
**women**								
35–44	8.7 (5.7–12.7)	3.8 (2.0–6.4)	8.1 (5.1–11.8)	2.9 (1.4–5.3)	3.3 (1.5–6.1)	0.6 (0.1–2.1)	0.0 (0.0–1.3)	0.6 (0.1–2.1)
45–54	**18.1 (13.9–22.9)**	**8.1 (5.4–11.5)**	6.6 (4.1–10.0)	5.2 (3.1–8.1)	6.3 (3.8–9.6)	1.4 (0.5–3.3)	3.9 (2.1–6.8)	1.7 (0.6–3.7)
55–64	**28.7 (25.5–34.3)**	**14.7 (11.0–19.1)**	6.6 (4.0–10.2)	6.6 (4.1–9.9)	10.1 (6.9–14.2)	9.4 (6.4–13.2)	8.4 (5.4–12.2)	4.7 (2.7–7.6)
65–74	**26.5 (20.2–33.6)**	**19.2 (14.8–24.3)**	5.5 (2.7–9.9)	13.6 (9.9–18.2)	17.1 (11.9–23.4)	11.2 (7.8–15.4)	11.6 (7.3–17.2)	5.2 (3.0–8.5)
75–79	12.2 (3.4–28.2)	14.0 (7.4–23.1)	9.1 (1.9–24.3)	18.6 (11.0–28.4)	27.3 (13.3–45.5)	9.3 (4.1–17.5)	12.1 (3.4–28.2)	10.5 (4.9–18.9)
35–79	**19.7 (17.4–22.2)**	**11.2 (9.6–13.0)**	6.8 (5.4–8.5)	7.5 (6.2–9.1)	**9.0 (7.3–10.8)**	**5.6 (4.4–6.9)**	5.6 (4.3–7.2)	3.4 (2.5–4.5)

*The sample was restricted to participants without known diabetes and to participants who were fasting for ≥8 hours. Data of 2,557 participants with normal glucose tolerance were included but not shown (SHIP: n = 991; KORA: n = 1,618). Results are crude prevalences(%) and 95% confidence intervals if not otherwise specified. Significant regional differences (p<0.05) are highlighted in bold. Abbreviations: SHIP-TREND: Study of Health in Pomerania (2008–2012); KORA F4: Cooperative Health Research in the Region of Augsburg (2006–2008); i-IFG: isolated impaired fasting glucose: 5.6–6.9 mmol/l (100–125 mg/dl, fasting); i-IGT: isolated impaired glucose tolerance: 7.8–11.0 mmol/l (140–199 mg/dl, 2 hour postload).

Finally, the odds of having unknown diabetes or prediabetes were about twofold increased in the northeast in comparison to the south (univariate OR: unknown diabetes vs. NGT: 2.12, 1.67–2.82; univariate OR: prediabetes vs. NGT: 1.78, 1.57–2.01). The association of glucose disorders with region was not explained by traditional risk factors and became even stronger after controlling for age, sex, BMI, physical activity, smoking, alcohol consumption and education. (OR_adj._: unknown diabetes vs. NGT: 2.59, 1.84–3.65; OR_adj._: prediabetes vs. NGT: 1.98, 1.70–2.31).

## Discussion

Our study shows that age-sex-standardized prevalence of unknown diabetes is considerably higher in the northeast than in the south of Germany (age group 35–79 years). Regional differences in prediabetes followed a similar northeast-south gradient and were more pronounced in the younger (35–59 years) than in the older age group (60–79 years). Thus, the prevalence of prediabetes in individuals from the northeast was largely comparable with that from individuals from the south, who were about 10 (women) to 20 years (men) older. Overall, regional differences were not explained by traditional risk factors such as BMI, physical activity, smoking, or low education.

This regional variation is in line with previous data on known type 2 diabetes prevalence in five regional population-based studies (DIAB-CORE), in nationwide health insurance data, and in a nationwide telephone survey (GEDA 2010) [4], [12], [13]. The increased prevalence of prediabetes and undiagnosed diabetes in the northeast in comparison to the south suggests that different diabetes screening activities are not likely to explain the regional differences in diabetes prevalence in Germany. Furthermore, a recent study based on the follow-up examinations in the DIAB-CORE consortium found that type 2 diabetes incidence (per 1,000 person-years) was also higher in the northeast than in the south of Germany (northeast: 13.2 (95%CI 10.9–16.1); south: 9.3 (7.7–11.3)) [14]. The present finding of a higher (pre)diabetes risk is in line with earlier reports on increased waist circumference levels, higher blood pressure and an increased (cardiovascular) mortality in Northeastern than in Southern Germany [15]–[17].

Previous results from our DIAB-CORE consortium suggested that regional deprivation, in particular the unemployment rate on municipality and neighborhood level partially explained the regional differences in known type 2 diabetes [18], [19]. These relationships were independent of obesity or individual socioeconomic status [18], [19]. Regional deprivation may also contribute to the regional differences in unknown diabetes and prediabetes. In this context, it is conceivable that relocation of healthier inhabitants to wealthier regions, which offer more jobs and a better overall infrastructure, may leave behind inhabitants with a generally higher morbidity and less healthy lifestyle.

It might also be of importance that the two study areas have been part of two different states during the years 1949–1990. The northeastern region of SHIP-TREND was part of the German Democratic Republic (GDR) and the southern region of KORA F4 was part of the Federal Republic of Germany (FRG). Both states differed considerably in their legislation, governmental, health care, and economic systems. Almost 25 years after reunification, persisting diversities have been reported which might be relevant for the different (pre)diabetes risk. First, in 1998, nutrition still differed between both regions [20]. As an example, tea, coffee and drinking water consumption was significantly lower in the former GDR than in the former FRG states, while the situation was inverse for soft drinks [20]. Furthermore, fruits were consumed more frequently in the territories of the former GDR, while leafy greens and other vegetables were predominantly consumed in the FRG [20]. Similar nutritional differences were found in a comparison of the EPIC-Potsdam (northeast) with the EPIC-Heidelberg (southwest) study [21]. Second, fetal and infant growth plays a role in the development of type 2 diabetes [22]. During the first years after reunification, combined statistics showed that perinatal mortality was increased in the territories of the former GDR in comparison to the federal states of the FRG [23]. It is conceivable that the perinatal mortality was also increased in the Northeast during the time when the study population of SHIP-TREND was born [23]. However, since 2000, perinatal mortality has declined in both regions and is currently lower in the territories of the former GDR. It is noteworthy, that a recent analysis of the German Perinatal Survey (2007–2011) reported a lower body length at birth for the neonates in the territories of the former GDR while there was no clear geographic pattern for preterm birth rate, birth weight, or the percentage of neonates with birth weight ≥4,500 g [24]. However, whether infant growth patterns, pancreas and liver development, may contribute to the regional differences in diabetes and prediabetes remains unclear.

There are few studies available on regional differences in diabetes prevalence from other countries. In China, regional differences in known and unknown type 2 diabetes seemed to arise from differences in urban and rural lifestyles [25]. While type 2 diabetes (known and unknown combined) was more frequent in urban areas (6.9% vs. 5.6%), the proportion of undiagnosed type 2 diabetes in total diabetes was higher in rural areas (70.5% vs. 58.0%). Urban-rural differences might not be relevant in our study because both regions have a similar geographic distribution of towns and rural areas. Therefore, novel risk factors for diabetes such as traffic-related air pollution (e.g. particle pollution, nitrogen oxides), job strain, emotional stress, anxiety or depressive disorders and the potential mediating role of inflammation need to be considered in future studies [26]–[29].

Regional differences in undiagnosed (pre)diabetes have important implications for health care planning. A meta-analysis demonstrated that individuals with prediabetes were 5–10 times more likely to develop diabetes annually than normoglycaemic individuals [30]. It has been estimated that up to 70% of individuals with prediabetes will develop diabetes over the course of life [31]. Furthermore, undiagnosed diabetes is associated with an increased mortality [32], [33]. In individuals with prediabetes an increased risk for neuropathy, nephropathy, retinopathy, and macrovascular disease has been described [31], [34]. In line with these observations, drug treatment costs were already increased in persons with prediabetes and unknown type 2 diabetes, when compared to individuals with normal glucose tolerance [35].

From a public health perspective, diabetes prevention strategies should take into account these regional characteristics [36], [37]. Our results indicate that type 2 diabetes prevention in Germany potentially needs to start at fairly young age (at least before the age of 35) in the northeast, whereas prevention in the south – especially in females - may possibly focus more on middle-aged populations.

Some limitations of the study have to be mentioned. First, in SHIP-TREND plasma glucose has been examined, while in KORA F4 serum glucose was analyzed. However, fasting plasma glucose and fasting serum glucose were compared in SHIP-TREND showing a high agreement between both methods. Second, KORA F4 is a follow-up survey and examinations have been carried out seven years after baseline examinations. At baseline, OGTTs were administered to all participants aged 55 years and older. Thus, exclusion of individuals with type 2 diabetes in this age group encompassed unknown diabetes and known diabetes, while only persons with known diabetes were excluded in SHIP-TREND. This might have influenced the results in older age groups. Therefore, we report results for younger and older participants separately. Furthermore, the SHIP-TREND study started examinations in 2008 (lasting until 2012) when the examinations in KORA F4 were already finished. The difference of four years on average in data collection might have influenced the results, however, probably only to a small degree.

The strength of the study is the large number of well-characterized participants that underwent OGTTs following common standardized protocols.

In conclusion, men and women in the northeast of Germany have a considerably higher prevalence of prediabetes (IFG, IGT or both) and unknown type 2 diabetes than their counterparts in the south of Germany. In particular, prediabetes is more prevalent at younger age in the northeast. Therefore, prevention strategies and health care policy need to take into account these regional differences in morbidity and diabetes risk. Further studies are warranted to examine the risk factors underlying these regional differences, in particular, the impact of individual and regional socioeconomic indicators.

## Supporting Information

Table S1 Characteristics of excluded participants with type 2 diabetes in SHIP-TREND and KORA F4*. *Results are means (SD), proportions (%) or Median (IQR). P-values were calculated using Wilcoxon-Test for metric and fisher exact test for dichotomous variables. Missing values in KORA: 14 for fasting glucose, 5 in BMI, 1 for BP and lipids. Missing values in SHIP-TREND: 1 for FPG and BMI, 3 for BP and lipids. Abbreviations: SHIP-TREND: Study of Health in Pomerania (2008–2012); KORA F4: Cooperative Health Research in the Region of Augsburg (2006–2008).
(DOCX)
