# Supplementary material for: Regional Differences of Undiagnosed Type 2 Diabetes and Prediabetes Prevalence Are Not Explained by Known Risk Factors
Source: PLoS One. 2014 Nov 17;9(11):e113154. doi: 10.1371/journal.pone.0113154 (PMC4234669; doi:10.1371/journal.pone.0113154)
Supplement: Table S1 — Characteristics of excluded participants with type 2 diabetes in SHIP-TREND and KORA F4*. *Results are means (SD), proportions (%) or Median (IQR). P-values were calculated using Wilcoxon-Test for metric and fisher exact test for dichotomous variables. Missing values in KORA: 14 for fasting glucose, 5 in BMI, 1 for BP and lipids. Missing values in SHIP-TREND: 1 for FPG and BMI, 3 for BP and lipids. Abbreviations: SHIP-TREND: Study of Health in Pomerania (2008–2012); KORA F4: Cooperative Health Research in the Region of Augsburg (2006–2008). (DOCX) [file pone.0113154.s001.docx]

**Supporting information**

Table S1 Characteristics of excluded participants with type 2 diabetes in SHIP-TREND and KORA F4

|  | **SHIP-TREND** | **KORA F4** | **p-Value** |
| --- | --- | --- | --- |
| N | 430 | 225 |  |
| Female sex (%) | 42.8 | 41.8 | 0.868 |
| Age (years) | 63.4 (10.2) | 66.9 (8.9) | <0.001 |
| Fasting glucose (mmol/l) | 8.7 (3.4) | 7.7 (2.1) | 0.006 |
| Body mass index(kg/m²) | 32.4 (5.9) | 31.3 (5.5) | 0.017 |
| Diastolic blood pressure (mmHg) | 78.1 (10.8) | 74.4 (10.6) | <0.001 |
| Systolic blood pressure (mmHg) | 137.4 (19.9) | 132.3 (19.8) | 0.001 |
| Total Cholesterol (mmol/l) | 5.1 (1.2) | 5.3 (1.1) | 0.072 |
| HDL-Cholesterol (mmol/l) | 1.3 (0.3) | 1.3 (0.3) | 0.279 |
| LDL-Cholesterol (mmol/l) | 3.0 (0.0) | 3.3 (0.9) | 0.011 |
| Triglycerides (mmol/l), Median (IQR) | 2.0 (1.4; 2.8) | 1.6 (1.1; 2.2) | <0.001 |
